# Quality evaluation of surimi and fish nuggets from Queen fish (*Scomberoides commersonnianus*)

**DOI:** 10.1002/fsn3.1172

**Published:** 2019-08-16

**Authors:** Marzieh Moosavi‐Nasab, Foroogh Asgari, Najme Oliyaei

**Affiliations:** ^1^ Seafood Processing Research Group, School of Agriculture Shiraz University Shiraz Iran; ^2^ Department of Food Science and Technology, School of Agriculture Shiraz University Shiraz Iran

**Keywords:** fish nugget, physicochemical properties, sensory evaluation, surimi nugget

## Abstract

The purpose of this study was formulation and evaluation of physicochemical properties of fish and surimi nuggets prepared from Queen fish (*Scomberiodes commersonnianus*) during 90 days of storage. Chemical analysis showed that surimi nuggets contained less protein, fat, and ash due to washing steps during surimi preparation. The titratable acidity, peroxide, and TBA values for fish nuggets were significantly higher than surimi nuggets during frozen storage (*p* < .05). Moreover, the textural properties of the products exhibited more firmness of surimi nuggets before cooking compared with fish nuggets (*p* < .05) and more firmness of fish nuggets after cooking compared with surimi one (*p* < .05). Furthermore, surimi nuggets were lighter and had lower total bacterial counts rather than fish nuggets during frozen storage (*p* < .05). SDS‐PAGE of the samples during storage exhibited more intensity of the bands related to α‐actinin, actin, and β‐tropomyosin in surimi nuggets compared with that for fish nuggets. Moreover, the sensory evaluation showed that acceptability of surimi nuggets was more than that for fish nuggets after frozen storage. These results showed that surimi nuggets had higher quality indicators rather than fish nuggets.

## 1 INTRODUCTION

Surimi is the odorless and white stabilized myofibrillar protein paste prepared from deboned fish flesh during the several washing process to remove the lipids and undesirable substances (Priyadarshini, Xavier, Nayak, Dhanapal, & Balange, 2017). Surimi has good gel‐forming ability which is used for manufacture of high‐quality and value‐added seafood products (Moreno, Herranz, Pérez‐Mateos, Sánchez‐Alonso, & Borderías, 2016). Type and condition of washing process during the surimi production plays an important role on quality of surimi‐based products because of an effective removal of the proteolytic enzymes and lipids (Priyadarshini et al., 2017). However, other parameters such as fish species, protein content, pH, and temperature have an influence on rheological properties of surimi (Panpipat, Chaijan, & Benjakul, 2010).

In addition, as its unique functional characteristics, several studies have been conducted to production of surimi from different fish species and treatments (Moon, Yoon, & Park, 2017; Panpipat et al., 2010; Priyadarshini et al., 2017; Yin & Park, 2015). Although, due to the increasing consumer nutritional requirements, the food industry is trying to manufacture the varieties of products from by‐product or less valuable fishes while still maintaining their desirable sensorial characteristics (Ali, Mansour, E‐lBedawey, & Osheba, 2017).

Further, there is a growing interest to find ways to innovative and nutritive foods. Thus, during the last years, many researches have been subjected to prepare and evaluation of textural and sensorial properties of fish burger and nuggets from deep flounder (Mahmoudzadeh, Motallebi, Hosseini, Khaksar, et al., 2010), tilapia (Bainy, Bertan, Corazza, & Lenzi, 2015), catla (Vanitha, Dhanapal, & Reddy, 2015), panguscat fish (Ejaz, Shikha, & Hossain, 2013), and grass crap (Haq, Dutta, Sultana, & Rahman, 2013). Moreover, utilization of surimi in food products is a way for production of value‐added aquatic foods (Y. Liu et al., 2014) and manufacturing of the processed fish or surimi‐based products with suitable quality properties and shelf life has been constantly increased during the last years (Coton, Denis, Cadot, & Coton, 2011). Moreover, it is of interest to expand the applications of surimi in food processing to further enhance the functionality of food properties. Thus, the development of fish or surimi nuggets can help to improve the fish consumption and manufacture of health food products.

Genus *Scomberoides* of the family Carangidae, a dominant finfish group found in Persian Gulf and the Oman sea, named “queen fish” (Masoomizadeh, Pazooki, & Valinassab, 2018). *S. commersonnianus* is one of the three species of genus *Scomberoides* that widely distributed throughout the Indo‐West Pacific (Panhwar, Qamar, & Jahangir, 2014). Queen fish usually consumed in fresh, frozen, dried, and salted form and the low cost, texture, and tasty flavor, make it appropriate for fish‐based products (Jamshidi & Shabanpour, 2013).

The aim of the present study was to preparation of surimi from *S. commersonnianus* and used for nugget formulation. In addition, we aimed to determine the physicochemical and sensorial properties of fish and surimi nuggets during 90 days of storage.

## 2 MATERIALS AND METHODS

### 2.1 Materials

*Scomberoides commersonnianus* was purchase from local market (Fars, Iran). Milk powder from kalleh company (Amol, Iran) and smoke powder from Saziba company (Tehran, Iran). Standard protein (10–200 KDa MW) from Thermo Scientific Fermentas (Canada). Spices were obtained from local market. All chemical materials were supplied from Merck (German).

### 2.2 Methods

#### 2.2.1 Surimi preparation from *S. commersonnianus*

Surimi was prepared using the method of Moosavi‐Nasab, Alli, Ismail, and Ngadi (2005) with some modifications. Frozen minced *S. commersonnianus* was obtained from local market (Shiraz, Iran). A quantity of frozen fish was thawed overnight at 4°C and washed (10 min) with chilled water using a 1:4 (w/v) ratio of minced to water. The washed mince was subjected to dewatering by covering with cheesecloth. The washing procedure was repeated three times. Appropriate quantities of sodium chloride (0.2% NaCl) were incorporated by blending with the mince.

#### 2.2.2 Nugget preparation from surimi

Two formulations were supplied for nuggets preparation. According to the following formulations (Table 1), surimi nuggets were contained the surimi (80.24%), water (9.95%), lemon (0.30%), onion (6.01%), spices (0.36%), smoke powder (0.20%), phosphate (0.20%), milk powder (1.50%), and dextrose (0.25%). The materials from formulation were weight to provide nuggets and then the mixture stored overnight (−20°C). Prior to coating, the nuggets were dusted with flour and then stored at −3°C until further analysis.

**Table 1:** Composition of surimi and fish nuggets formulas

Ingredients (%)	Surimi nuggets	Fish nuggets
Surimi	80.24	–
Fish minced meat	–	80.24
Water	9.95	9.95
lemon	0.30	0.30
Onion	6.01	6.01
Salt	0.99	0.99
Spices	0.36	0.36
Phosphate	0.20	0.20
Dextrose	0.25	0.25
Milk powder	1.50	1.50
Smoke powder	0.20	0.20

#### 2.2.3 Nugget preparation from fish minced meat

Fish nuggets were produced as above described with fish minced meat instead of surimi. About 1 Kg fillets were minced and mixed with ingredients according to Table 1, then stored overnight (−20°C). Flour was used as the dust and stored at −3°C.

#### 2.2.4 Proximate compositions

Determination of nuggets composition (moisture, lipid, protein, and ash) was carried out according to the AOAC (1995). Moisture content was determined using an oven. Kjeldahl and Soxhlet–Henkel methods were used for the determination of total protein (crud protein, *N* = 6.25) and fat content respectively. Also, ash content was measured by mineralization at 550°C.

#### 2.2.5 pH and titratable acidity measurement

The pH was measured for the homogeneous mixtures of nugget and distilled water (1:4, w:v) at the first and end of the storage using pH meter (PHT‐110, LABTRON, Iran). Titratable acidity was measured as by titration to neutrality with 0.1 N NaOH and calculated as ml of 0.1 N NaOH/g sample (Capita, Llorente‐Marigomez, Prieto, & Alonso‐Calleja, 2006).

#### 2.2.6 Peroxide value (PV)

PV of nuggets was calculated with the method of AOCS (1997). The sample (3 g) was heated in a water bath (60°C for 3 min), then thoroughly agitated for 3 min with 30 ml of acetic acid–chloroform solution (3:2 v/v), followed by the addition of saturated potassium iodide solution (1 ml). The reaction mixture was allowed to stand in the dark for 5 min and then was titrated with standard solution of sodium thiosulfate (25 g/L). The PV was calculated as meq/kg sample using the following equation:

$$\text{PV}\left(\text{meq}/\text{kg}\right)=\left(\text{S}\times \text{N}\right)/\text{kg}\times 100$$

where S is the volume of titration (ml), N the normality of sodium thiosulfate solution (*N* = 0.01), and W the sample weight (kg).

#### 2.2.7 Thiobarbituric acid (TBA) value

Thiobarbituric acid value of surimi and fish nuggets was performed as described by Sallam, Ishioroshi, and Samejima (2004) with slight modification. A stock solution containing 0.37% TBA, 15% TCA, and 0.25 N HCl was slowly heated to 75°C in water bath to facilitate the dissolution of thiobarbituric acid. Two ml of this solution mixed with 1 ml of homogenized sample, and then, the mixture was heated for 15 min in a boiling water bath to develop pink color. After cooling by tap water and centrifuging at 2000 × *g* for 15 min, the absorbance of the supernatant was determined spectrophotometrically (model UNICO UV‐2100 Spectrophotometer) at 532 nm. The TBA value was expressed as mg malondialdehyde/kg of nugget.

#### 2.2.8 Nugget texture analysis

Texture profile analysis (TPA) of nuggets was measured before and after cooking by the method described by C. Cardoso, Mendes, and Nunes (2008) using a Texture Analyser (Texture Pro CT V1.3 Buil 15). Chilled and fried samples were tempered to bring to the room temperature (25°C). The nuggets were cut into uniform‐sized pieces (6 × 3.5 × 1 cm) and placed on the sample holder. Then, puncture test was carried out by penetrating the sample to breaking point with metal probe equipped with 6‐mm‐diameter spherical head using the speed of 6 mm/s. Finally, breaking force (N) and breaking deformation (mm) were evaluated. Three measurements were taken from each sample and averaged for statistical analysis.

#### 2.2.9 Color analysis

Color measurements of nuggets were obtained using a colorimeter chamber. Color parameters (L*, a* and b*) were gained from different spots on surface of each sample using Photoshop software (CS3; Nguyen & Hwang, 2016).

#### 2.2.10 Sensory assessment

Sensory evaluation of nuggets was conducted using twelve assessors who trained prior to the experiment, using a 5‐point hedonic scale (5 = like extremely, 1 = dislike extremely) following the method by Carpenter, O’grady, O’callaghan, O’brien, and Kerry (2007). Sensory assessment for various quality attributes of each fried nuggets such as taste, aroma, texture, color, and overall acceptability was recorded. Nuggets were fried, and the assessors were then served with slice of nugget presented in individual booths under clear white fluorescent light together with cold water to clean the palate between samples. The Descriptors were rated on a scale from “1” representing the lowest score and “5” the highest one. The assessors were demanded to appraise the nuggets quality by scoring for the parameters.

#### 2.2.11 Sodium dodecyl sulfate‐polyacrylamide gel electrophoresis (SDS‐PAGE)

The freeze‐dried nuggets were analyzed for protein composition and molecular weight using SDS‐PAGE, as described by Laemmli (1970). The samples (20 μg) were mixed (1:1) with a sample buffer containing dithiothreitol, heated for 5 min in a boiling water bath, then loaded into a 1.5‐mm acrylamide gel slab (10% T) assembled in a vertical electrophoresis unit. After electrophoresis, the gels were stained for 2 hr with a solution containing 0.5% Coomassie Brilliant Blue R‐250, 40% methanol, and 7% acetic acid. The excess stain was removed with a solution containing 40% methanol and 7% acetic. The molecular weights of samples were estimated by reference to the relative mobilities of standard proteins.

#### 2.2.12 Microbiological analysis

To determine the total plate count (TPC) for each sample, applied the spread plate method using Plate Count Agar. The average number of colonies for each sample was expressed as log_10_ cfu/g sample (AOAC, 1995).

### 2.3 Statistical analysis

Data were analyzed by SPSS 19 with ANOVA and Duncan's multiple range test for mean comparison. All analyses were carried out in triplicate at least. Significance of differences was defined as the 5% level (*p* < .05).

## 3 RESULTS AND DISCUSSION

### 3.1 Proximate composition

Table 2 indicates the chemical composition of nuggets at the first day. According to the results, surimi nuggets had the lower moisture, protein, and lipid content rather than fish nuggets significantly (*p* < .05). The lower protein content of surimi nuggets was probably due to the loss of protein during the washing process of surimi production. Because water soluble sarcoplasmic proteins leached out minced fish, moreover, myofibrillar proteins can become soluble in three times washing and then lost, resulting in a lower yield of protein recovery (Karthikeyan, Dileep, & Shamasundar, 2006; Rawdkuen, Sai‐Ut, Khamsorn, Chaijan, & Benjakul, 2009). The times of washing cycle would be an effective means to decrease the proteins and pigments (Chaijan, Benjakul, Visessanguan, & Faustman, 2004). Furthermore, surimi nuggets had lower amount of ash (2.77%) rather than fish nuggets (3.84%) which can be attributed to the leaching out of the water‐soluble components during washing process of surimi. The proximate compositions of surimi are critical factors impact on high quality of surimi products. Protein concentration extremely impacts on the gel properties. Also, low lipid content causes prolongation of the shelf life of product and inhibited the lipid oxidation (Jin et al., 2007). This finding also was agreed with Coelho, Weschenfelder, Meinert, Amboni, and Beirão (2007) who found Hake (*Merluccius hubsi*) surimi had lower moisture and protein content rather than Hake (*Merluccius hubsi*) fish as results of three washing processes not only due to the removing of sarcoplasmic proteins, but also for covering effect of starch and flour and diluting the proteins in burger formulation. The reduction in proximate compositions of fish fingers produced from washed mince was attributed to washing treatment (Tokur, Ozkütük, Atici, Ozyurt, & Ozyurt, 2006).

**Table 2:** Proximate composition of nuggets stored at −20°C

Sample	Moisture (%)	Protein (dw%)	Lipid (dw%)	Ash (dw%)
Surimi nuggets	67.09 ± 0.28^a^	16.97 ± 0.64^a^	0.43 ± 0.04^a^	2.77 ± 0.15^a^
Fish nuggets	69.60 ± 0.45^b^	18.32 ± 0.13^b^	0.55 ± 0.03^b^	3.84 ± 0.12^b^

Means with different letters are significantly different (*p* < .05). Each value is expressed as Mean ± *SD*, and test was conducted in triplicate.

### 3.2 pH and titratable acidity

The results of pH and titratable acidity of nuggets are shown in Table 3. As can be seen, fish nuggets had higher titratable acidity rather than surimi nuggets (*p* < .05). In addition, the acidity of two nugget formulations also increased throughout the whole storage period (*p* < .05). Generally, the nuggets prepared with surimi had lower acidity significantly at the end of storage period (*p* < .05), whereas, higher titratable acidity was observed in fish nuggets. In addition, there were no significant differences between pH of both nugget samples. The initial pH in surimi and fish nuggets was 7.40 and 7.35, respectively. However, storage had a significant effect on pH value and a reduction was observed in pH of samples throughout the storage (*p* < .05). During the whole storage period, the pH reached to 6.85 in surimi nuggets, while it was 6.75 in fish nugget. The reduction in pH might be related to the fermentation of some ingredient or due to the addition of spices (Vanitha et al., 2015). Additionally, the reduction in oxygen and enhancement of CO_2_ content because of aerobic microflora growth might cause pH decline after three months (Mahmoudzadeh, Motallebi, Hosseini, Khaksar, et al., 2010). The pH value around 6.8–7 is acceptance limit of fish meet and higher than 7 is considered to be spoiled. Although, pH value is not reliable indicator of quality control (Mahmoudzadeh, Motallebi, Hosseini, Haratian, et al., 2010). However, our results were in agreement with Haq et al. (2013) who obtained the pH of fish burger from grass carp about 6.60 and claimed the burger produced from mince with around neutral pH had appropriate quality attributes. In addition, Ejaz et al. (2013) obtained similar observation in the pH (6.6 ± 0.05) of pangus catfish burger.

**Table 3:** pH, titratable acidity, and PV of surimi and fish nuggets during storage

Storage (day)	pH		Titratable acidity (mg/g)		PV (meq/kg)	
	Surimi nuggets	Fish Nugget	Surimi Nuggets	Fish nuggets	Surimi nuggets	Fish nuggets
0	7.40 ± 0.48^Aa^	7.35 ± 0.34^Aa^	1.24 ± 0.01^Be^	1.34 ± 0.01^Ae^	0.70 ± 0.02^Be^	1.54 ± 0.01^Ae^
1	‐	‐	2.53 ± 0.02^Bd^	3.63 ± 0.04^Ad^	1.15 ± 0.01^Bd^	2.35 ± 0.02^Ad^
14	‐	‐	3.65 ± 0.03^Bc^	4.63 ± 0.01^Ac^	1.65 ± 0.01^Bc^	3.74 ± 0.02^Ac^
30	‐	‐	8.45 ± 0.02^Bb^	9.34 ± 0.01^Ab^	1.75 ± 0.01^Bb^	3.86 ± 0.02^Ab^
90	6.85 ± 0.3^Ab^	6.75 ± 0.14^Ab^	10.10 ± 0.02^Ba^	11.67 ± 0.02^Aa^	2.50 ± 0.04^Ba^	4.34 ± 0.01^Aa^

Means with different capital letters in each row and small letters in each column are significantly different (*p* < .05). Each value is expressed as Mean ± *SD*, and test was conducted in triplicate.

### 3.3 PV

Table 3 shows the changes in PV of nuggets during storage at −20°C. Results revealed that the PV of samples tended to enhance with storage period significantly (*p* < .05) and from the initial PV of 0.70 (meq/kg) in surimi nuggets and 1.54 (meq/kg) in fish nuggets reached to 2.50 and 4.34 (meq/kg) after 90 days respectively. This results indicated the hydroperoxides formation as primary lipid oxidation products (Hwang et al., 2011). Moreover, significantly higher PV was noted in fish nuggets in comparison with surimi one (*p* < .05). The same observation was also obtained by Vanitha et al. (2015) during the refrigerated studies of fish burger from catla with increasing PV to 7.28 meq/kg after 5 days of storage. Tokur, Polat, Beklevik, and Özkütük (2004) also observed a significant increase in PV of tilapia fish burger during storage. Similar results were obtained by Ninan, Bindu, and Joseph (2010) who reported that in fish cutlet, PV exhibited an increase during the twelve‐week frozen storage. As mentioned before, the increasing in the PV could be related to the penetration of oxygen in the minced meat and accelerated the lipid oxidation (Vanitha, Dhanapal, Sravani, & Reddy, 2013). Moreover, as can be seen, addition of surimi causes lower PV rather than fish nuggets. This might be attributed to lower lipid content of surimi and consequently in surimi nuggets.

### 3.4 TBA value

Lipid oxidation is an important factor for spoilage in frozen fish and fishery products and can negatively affect protein functionality, also causes discoloration, off‐odor, and off‐flavor in products (Al‐Hijazeen, Lee, Mendonca, & Ahn, 2016). The TBA as measurement of secondary lipid oxidation products was calculated at 1st and 90th day of storage, and the results are presented in Table 4. The lower TBA value was observed in the product consisted of surimi (0.02 mg malondialdehyde/kg) compared with fish nuggets (*p* < .05). In addition, the enhancing of TBA values with the prolongation of storage period was observed (*p* < .05). At the beginning of the storage, TBA values were determined as 0.02 ± 0.00 (mg malondialdehyde/kg) and 0.03 ± 0.00 (mg malondialdehyde/kg) for surimi and fish nuggets, respectively. TBA value for both nugget formulas increased to 0.04 ± 0.00 (mg malondialdehyde/kg) and 0.05 ± 0.00 (mg malondialdehyde/kg), respectively, as the storage time increased (*p* < .05). TBA in an important indicator for fish and fish product quality. It seem the washing process of surimi production decreased the TBA value due to removing the considerable amount of lipid (G. R. Shaviklo, Thorkelsson, Arason, Kristinsson, & Sveinsdottir, 2010). The same result was found in Tokur et al. (2006) who reported the enhancement of the TBA value in the unwashed mince mirror carb fingers compare to the washed sample. They claimed that washing treatment had impact on TBA value. Tokur et al. (2004) also observed an increase in the TBA value of fish burger from tilapia (*Oreochromis niloticus*) after eight months. In addition, the similar trends in the TBA values of nile tilapia fish burger (Bahar, Abdurrahman, Gulsun, & Serhat, 2004), tilapia fish cutlet (Ninan et al., 2010), grass carp fish cutlet and fish finger (Pandey & Kulkarni, 2007) and nile tilapia nugget (Lima et al., 2015) were obtained. The increase in TBA might be related to the oxygen availability for oxidation which is attributed to mechanical mincing of fish meat or mixing of ingredients (Tokur et al., 2004) or might be attributed to the packaging. TBA value is an index of lipid oxidation in meat products due to aldehydes and carbonyls production from hydrocarbons and the rancid flavor is initially detected in meat products between TBA values of 0.5 and 2.0 (Sallam et al., 2004) which our results were lower than this range.

**Table 4:** TBA of nuggets at 1st and 90th day of storage

Sample	Day	TBA (mg malondialdehyde/kg)
Surimi nuggets	1st	0.02 ± 0.00^Ab^
	90th	0.04 ± 0.00^Aa^
Fish nuggets	1st	0.03 ± 0.00^Bb^
	90th	0.05 ± 0.00^Ba^

Means with different capital letters in each row and small letters in each column are significantly different (*p* < .05). Each value is expressed as Mean ± *SD*, and test was conducted in triplicate.

### 3.5 TPA

Table 5 shows the TPA results of nuggets before and after cooking. As can be seen, the hardness exhibited a significant difference between surimi and fish nuggets before cooking (*p* < .05) and the highest breaking force (3.87 ± 0.35 N/mm) was found in surimi nuggets after 90 days of storage. The results indicated that the surimi increased the tenderness and improved the nugget texture. These results were attributed to the integrity of proteins in surimi, especially myofibrillar proteins because of their ATPas activities which generally used as a measurement of actomyosin integrity. Moreover, the sarcoplasmic proteins influenced on strength of surimi gel (Panpipat et al., 2010). This may also had been the reason for the lower lipid content of surimi compared with fish, as Tolasa, Lee, and Cakli (2010) evaluated the textural properties of surimi fortified with omega 3 and observed the penetration force decreased by increasing the oil content. Thus, a little higher hardness of surimi nugget could be attributed to the lower lipid content of surimi rather than fish nugget. Further, the changes of breaking force in both formulations during the storage was greatly and increased with prolongation of storage period (*p* < .05). The hardness of fresh surimi and fish nugget was 0.38 ± 0.01 and 0.24 ± 0.01 (N/mm), respectively, which enhanced to 3.87 ± 0.35 and 1.60 ± 0.01 (N/mm) at the end of storage (*p* < .05). The same trend was observed in hardness of cooked samples during storage, and the results exhibited that a significant increase in the breaking force after cooking in all treatments. However, fish nuggets showed higher hardness compared with the other (*p* < .05). The highest observation of hardness was related to fish nuggets (5.44 ± 0.02 N/mm) at 90th day. The improvement of texture attribute might be affected by heating because the thermal treatments changed the quality of surimi products. Surimi gelation process and formation of three‐dimensional network structures occurred about 40°C and gel strength enhanced with heating time increasing (C. L. Cardoso, Mendes, Vaz‐Pires, & Nunes, 2012). In addition, expansion of myofibrillar proteins led to exposure of functional group such as sulfhydryl group. Therefore, cross‐linking interactions between –SH groups and formation of S‐S bond caused proteins got aggregated and the gel strength increased (Park, 2013; Xu, Xia, Yang, & Nie, 2010). Thus, the decrease in –SH content revealed the increase of S‐S bond which could be affected by type of heating. Therefore, fish nuggets exhibited higher hardness compared with surimi nugget because of lower protein content of surimi. Cao et al. (2018) studied the effect of combination of traditional water bath and microwave heating on surimi gel strength. They reported microwave heating during the second step of heating improved the gel strength which could be related to the fact that more cross‐linking S‐S bonds, consequently more compact network structure. Moreover, the effect of ingredient on nugget texture was evaluated by Chen, Chen, Chao, and Lin (2009) which reported addition of 1% wheat protein or soy protein caused desirable hardness and crispness in fried nugget. Further, Makri (2012) analyzed the effects of flour on textural parameters. Their research confirmed that fish nugget formulated with corn flour had lower hardness rather than formulations with wheat and potato flour.

**Table 5:** The hardness (N/mm) of nuggets before and after cooking during different times

Time (day)	Before cooking		After cooking	
	Surimi nuggets	Fish nuggets	Surimi nuggets	Fish nuggets
0	0.38 ± 0.01^Ae^	0.24 ± 0.01^Be^	1.93 ± 0.02^Ae^	1.50 ± 0.01^Be^
1	0.41 ± 0.01^Ad^	0.40 ± 0.01^Bd^	2.04 ± 0.01^Bd^	2.54 ± 0.01^Ad^
14	0.50 ± 0.01^Ac^	0.45 ± 0.02^Bc^	2.16 ± 0.01^Bc^	2.63 ± 0.01^Ac^
30	1.67 ± 0.01^Ab^	0.61 ± 0.01^Bb^	4.16 ± 0.01^Bb^	4.82 ± 0.01^Ab^
90	3.87 ± 0.35^Aa^	1.60 ± 0.01^Ba^	4.55 ± 0.01^Ba^	5.44 ± 0.02^Aa^

Means with different capital letters in each row and small letters in each column are significantly different (*p* < .05). Each value is expressed as Mean ± *SD*, and test was conducted in triplicate.

### 3.6 Color analysis

Colors expressed as L* value (lightness), a* value (redness), and b* value (yellowness) were analyzed during storage and are presented in Table 6. The lightness values of nuggets decreased with increasing storage time significantly and a sharp decline was observed at the end of storage. Whereas a slight decrease with no significant difference during the whole storage was obtained from redness and yellowness in both formulations. The results among samples showed that surimi nuggets were lighter than fish nuggets except at 90th day and had lower a* and b* values rather than fish nugget with no significant difference (*p* > .05). The lighter color of surimi nuggets could be related to the effective removal of myoglobin and hemoglobin as two major pigments from the muscle during the washing which was strongly influenced by the pH and NaCl concentration (Chaijan et al., 2004; Rawdkuen et al., 2009). Leaching process has a beneficial effect on color by increasing lightness and reducing redness (A. R. Shaviklo & Rafipour, 2013). Moreover, the reduction in redness might be related to oxymyoglobin formation as a result of protein oxidation (Chaijan et al., 2004). As expected fish nugget had higher a* value because of its higher lipid content and oxidation susceptibility. However, Al‐Bulushi, Kasapis, Al‐Oufi, and Al‐Mamari (2005) found that the lightness of fish burger prepared from arabian sea meager (*Argyrosomus heinii*) remained stable during storage period which was attributed to the white fleshed of arabian sea meager with low content of myoglobin. Priyadarshini et al. (2017) reported that washing with 0.2% CaCl_2_ could improve the lightness of surimi gel because of creation of insoluble particles with light scattering effect as a result of ionic interaction between CaCl_2_ and anion in muscle. Further, Makri (2012) evaluated the effect of different flours on sea bream fish burger color properties and claimed that addition of wheat and corn flours causes higher L* value, while potato flour resulted in lower lightness.

**Table 6:** Color parameters of surimi and fish nuggets during storage

Storage (day)	L*		a*		b*	
	Surimi nuggets	Fish Nuggets	Surimi nuggets	Fish nuggets	Surimi nuggets	Fish nuggets
0	62.30 ± 5.51^Aa^	58.30 ± 6.51^Aa^	10.00 ± 3.00^Aa^	11.00 ± 2.00^Aa^	50.67 ± 1.15^Aa^	52.33 ± 4.90^Aa^
1	57.30 ± 4.16^Aa^	52.30 ± 3.20^Aa^	10.00 ± 2.00^Aa^	12.67 ± 2.10^Aa^	49.33 ± 0.57^Aa^	51.34 ± 4.51^Aa^
14	57.10 ± 4.00^Aa^	49.30 ± 8.20^Aa^	9.87 ± 4.10^Aa^	9.27 ± 3.50^Aa^	46.00 ± 4.36^Aa^	47.23 ± 2.87^Aab^
30	57.00 ± 4.00^Aa^	49.00 ± 8.20^Aa^	8.00 ± 1.00^Aa^	9.00 ± 3.60^Aa^	39.14 ± 2.08^Bb^	43.00 ± 1.73^Ab^
90	29.30 ± 3.06^Ab^	31.70 ± 7.20^Ab^	8.30 ± 2.30^Aa^	10.30 ± 3.80^Aa^	36.67 ± 2.52^Bb^	40.62 ± 1.31^Ab^

Means with different capital letters in each row and small letters in each column are significantly different (*p* < .05). Each value is expressed as Mean ± *SD*, and test was conducted in triplicate.

### 3.7 Sensory evaluation

**Figure 1:**
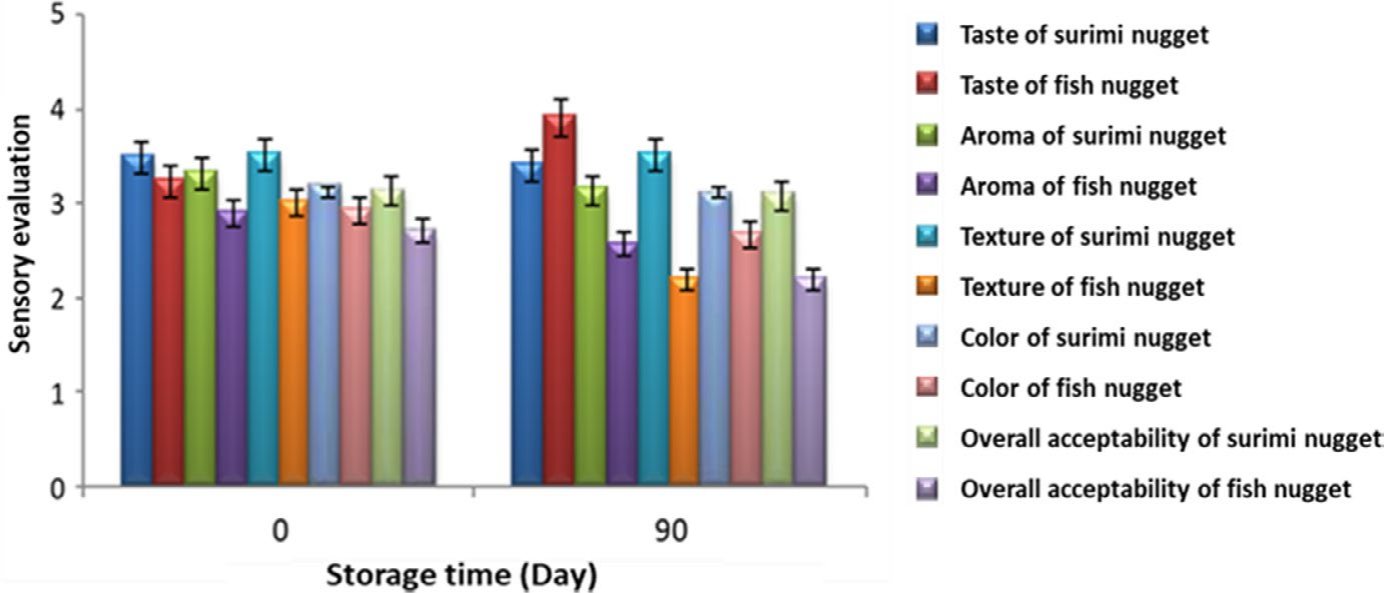
Sensorial evaluation of surimi and fish nuggets during storage

Sensory attributes of samples were evaluated on the first and last day of storage time (Figure 1). In general, the sensory scores given by the panel of judges to taste, aroma, texture, color, and overall acceptability varied significantly between the surimi and fish nuggets (*p* < .05). It seems, surimi nuggets had higher score rather than fish nuggets in all attributes on the first day. However, no significance difference was observed between aroma of surimi and fish nuggets at the end of storage (*p* > .05). Furthermore, no significant changes occurred in taste, aroma, texture, and color of surimi nuggets during storage while it is evident from Figure 1 that the sensory score of fish nugget given by the assessors decreased as the storage interval increased. Moreover, the texture of fish nugget got stiff and get lower score in aroma, color, and overall acceptability at the end of storage (*p* < .05). One of the main factors of effects on texture properties is water holding capacity (WHC). It can be seen, nuggets formulated with surimi had better texture score rather than fish nugget. This can be attributed to the better WHC of surimi gel network (Filomena‐Ambrosio et al., 2016). This phenomenon is related to washing with NaCl because chloride ions penetrate into the myofibrillar proteins and increase the electrostatic repulsion between filaments, thus increase the protein's affinity for water and enhance the entrapped water (Wang, Zhang, Bhandari, & Yang, 2018). Moreover, water attracted to negative charges of the myofibrillar proteins such as helical structure of myosin and increased the WHC. In addition, some additives such as phosphate resulting in the dissociation of the actomyosin into the actin and myosin. Thus phosphate played a prominent role in the properties of seafood by increasing the water retention in products (Filomena‐Ambrosio et al., 2016). The results also showed that nuggets made from surimi were favored by assessors because surimi nuggets scored higher for all attributes at the end of storage whereas the other one scored lower. It seems that the washing process of surimi production has crucial impact on myoglobin removal, color improvement, and gel strengthening of surimi (Jin et al., 2007); thus, surimi nuggets had better color and texture properties rather than fish nugget. This result is in agreement with the results of Vanitha et al. (2013) who obtained the high score for sensory properties of fish cutlet and fish burger after 90 days of storage.

### 3.8 SDS‐PAGE

**Figure 2:**
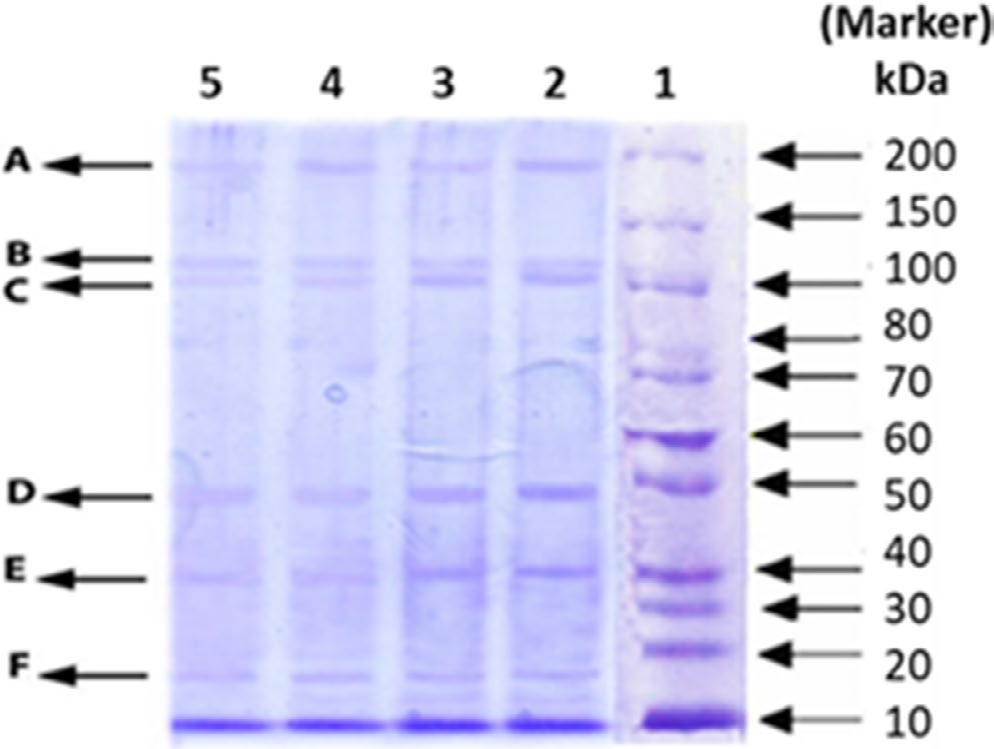
SDS‐PAGE patterns of proteins in nuggets at 0 and 90th day of storage at −20°C. Column 1: marker, columns 2 and 3: surimi nuggets, and columns 4 and 5: fish nuggets at 0 and after 90 days of storage, respectively

Figure 2 illustrates the electrophoretic pattern of surimi and fish nuggets to explore the pattern of molecular weight of proteins during storage at −20°C. Moreover, the probable identification of molecular weight of proteins gained is shown in Table 7. According to the Figure 2, the similar bands between 200 and 100 kDa as well as low molecular weight bands between 50 and 20 kDa were obtained in both nugget formulations. As can be seen, no marked differences were observed in bands of surimi nuggets at the zero and 90th storage. Moreover, the similar trend was obtained in protein patterns of fish nugget. However, the intensity of bands between two samples was different and the intensity of α‐actinin, actin, and β‐tropomyosin bands were higher in surimi nuggets rather than fish nugget. As expected, more of sarcoplasmic proteins removed during the washing step in surimi production and consequently increased the myofibrillar proteins concentration such as actin. Thus, the intensity of the bands was higher in nugget prepared with surimi. At the end of storage, a slight reduction in the amount of myosin heavy chain (MHC, around 200 kDa) was observed. Nevertheless, it seems actin was more stable which is attributed to its interactions with myosin (Sun & Holley, 2011). The similar observation in protein pattern band was found in croaker surimi (Panpipat et al., 2010; Van Phu, Morioka, & Itoh, 2010), tilapia surimi (Rawdkuen et al., 2009), and silver crap muscle (R. Liu et al., 2010). However, it should be noticed that the different solutions have great influence on removing of sarcoplasmic proteins from minced fish as Priyadarshini et al. (2017) showed the effect of different washing solutions, alkaline saline, and calcium chloride with salt compared with unwashed mince which successfully removed soluble proteins.

**Table 7:** Molecular weight (MW) of proteins and probable identification of bands in SDS‐PAGE pattern

Probable identification	MW (kDa)	Band
Myosin heavy chain (MHC)	194.4	A
C‐protein	115.2	B
α‐actinin	102.6	C
Actin	46.8	D
β‐tropomyosin	39.5	E
Myosin light chain (MLC)	18.2	F

### 3.9 Microbiological analysis

The changes in the TPC of products during frozen storage were enumerated, and the results are presented in Table 8. A steady decrease in TPC from the initial value of 4.55 log_10_ cfu/g to 3.59 log_10_ cfu/g was observed in surimi nuggets stored at −20°C over a period of 90 days while the bacterial count of fish nugget was initially 4.59 log_10_ cfu/g which reached to 3.63 log_10_ cfu/g at the end of storage period (*p* < .05). In addition, a significant difference was observed in TPC of surimi and fish nuggets (*p* < .05). The hygiene condition of fish handling and surimi preparation had an important role on the initial microbial count of samples (A. R. Shaviklo & Rafipour, 2013). In addition, the reduction in microbial count of products could be attributed to the effect of freezing on preservation of growth and activity of microorganisms or powerful antimicrobial properties of spices used in products (Jamshidi & Shabanpour, 2013; Vanitha et al., 2013). For instance, the garlic's potential to destroy microorganisms is established (Al‐Bulushi et al., 2005). This result was in agreement with the finding of Al‐Bulushi et al., (2005) who reported the initial bacterial count for fish burger about 3 × 10^4^ cfu/g which indicate the high quality of raw material. Moreover, they obtain the reduction in aerobic bacterial count during storage at −20°C for three months that showed the hygienic condition of processing. However, the results are accepted as 10^7^ cfu/g is the maximum acceptable bacterial load for appropriate shelf life (Al‐Bulushi et al., 2005).

**Table 8:** Microbiological characteristics (log_10_ cfu/g) of nuggets during storage

Storage (Day)	Surimi nuggets	Fish nugget
0	4.55 ± 0.007 ^Ba^	4.59 ± 0.007^Aa^
90	3.59 ± 0.007 ^Bb^	3.63 ± 0.007^Ab^

Means with different capital letters in each row and small letters in each column are significantly different (*p* < .05). Each value is expressed as Mean ± *SD*, and test was conducted in triplicate.

## 4 CONCLUSION

In summary, in an effort for innovative utilization of surimi, we have successfully prepared surimi nuggets from *S. commersonnianus*. The monitoring of proximate composition of nuggets revealed that surimi nuggets contained less protein, fat, and ash due to washing steps during surimi preparation. The chemical parameters (titratable acidity, PV, and TBA) and TPA values of both samples increased with the storage period; however, surimi nugget showed better results. Although the increase in hardness of both nuggets was observed after cooking, but surimi had appropriate texture property. Moreover, the color attributes were decreased during the storage; however, the sensorial evaluation of samples exhibited that the surimi nugget had higher hedonic scores rather than fish nugget (*p* < .05). In addition, the SDS‐PAGE confirmed the similar bands in both nugget formulations. Further, the successful production of surimi nuggets had lower microbial count rather than fish nuggets. This study suggested that applying surimi with appropriate amount of ingredients formulation could develop an alternative ready‐to‐eat product from fish.

## CONFLICT OF INTEREST

We declare that we have no conflict of interest.

## ETHICAL STATEMENTS

This study does not involve any human or animal testing.
